# Quality of life and complications after nephron-sparing treatment of renal cell carcinoma stage T1—a systematic review

**DOI:** 10.1186/s13643-021-01868-2

**Published:** 2022-01-04

**Authors:** Theresa Junker, Louise Duus, Benjamin S. B. Rasmussen, Nessn Azawi, Lars Lund, Ole Graumann, Birgitte Nørgaard

**Affiliations:** 1 Department of Radiology, Odense University Hospital, Kløvervænget 10, Indgang 112, 5000 Odense C, Denmark; 2 Department of Clinical Research, University of Southern Denmark, Kløvervænget 10, Indgang 112, 5000 Odense C, Denmark; 3 Department of Urology, Odense University Hospital, J. B. Winsløws Vej 4, Odense C, 5000 Denmark; 4 Department of Urology, Zealand University Hospital, Sygehusvej 10, 4000 Roskilde, Denmark; 5 Institute of Clinical Medicine, University of Copenhagen, Sygehusvej 10, 4000 Roskilde, Denmark; 6 Department of Public Health, User Perspectives and Community-based Interventions, University of Southern Denmark, J.B. Winsløws Vej 9B, 5000 Odense C, Denmark

## Abstract

**Background:**

Despite the fact that nephron-sparing treatment is considered preferable from a surgical perspective patients’ quality of life (QoL) following different types of nephron-sparing treatments remains unclear.

**Purpose:**

To investigate the quality of life and complications after nephron-sparing treatment of renal cell carcinomas of stage T1.

**Materials and methods:**

A systematic search of six databases was carried out. We included studies that reported the quality of life and complications in patients aged 18 years or older following nephron-sparing treatment of renal cell carcinoma stage T1. The quality assessment was performed using the Critical Appraisal Skills Programme (CASP) checklist for cohort studies and the CASP Randomized Controlled Trial Checklist. Data were analyzed using a narrative approach.

**Results:**

Eight studies were included, six of which investigated QoL after partial nephrectomy and two after ablation therapies. Seven studies reported complications. Three studies reported higher QoL scores after partial nephrectomy compared to radical nephrectomy. Two studies showed that QoL increased or returned to baseline levels up to 12 months following partial nephrectomy. One study reported a gradual increase in QoL after radiofrequency ablation, and one study reported that all patients recovered to baseline QoL following cryoablation. Across studies, we found a complication rate up to 20% after partial nephrectomy and up to 12.5% after ablation therapy.

**Conclusions:**

The results of this systematic review suggest that nephron-sparing treatment appears to be superior or comparable to other treatment alternatives with regard to QoL outcomes. Additionally, based on the studies included in this review, partial nephrectomy appears to have a higher complication rate compared with ablation therapies.

**Systematic review registration:**

PROSPERO CRD42020155594

**Supplementary Information:**

The online version contains supplementary material available at 10.1186/s13643-021-01868-2.

## Introduction

The incidence of renal cell carcinoma (RCC) has increased worldwide and more than doubled in the USA since 1975 [1]. In particular, the detection of localized RCC has increased and is typically comprised of 20% benign tumors and about 20–25% potentially aggressive RCC at the time of diagnosis [2, 3]. Surgery is the only potentially curative treatment option [4]. Within the area of surgical treatment, the focus is on performing procedures that are as minimally invasive as possible, and preserving as much healthy renal tissue as possible, without compromising the oncological outcome [3, 4]. Since the increased incidence in RCC mainly involves tumors of stage T1, nephron-sparing approaches, such as partial nephrectomy (PN) and ablation therapy, which includes radiofrequency ablation (RFA), cryoablation (CA), and microwave ablation (MWA) have become more attractive [2]. According to the American Urological Association and the European Association of Urology guidelines, patients with stage T1 RCC should be offered nephron-sparing surgery (NSS) [4, 5]. Patients with stage T1 RCC stage are often without symptoms and the diagnosis frequently incidental [1]. Thus, the treatment alone carries a potential risk of negatively affecting patients’ quality of life (QoL), particularly if treatment leads to complications and/or confirmation of malignancy [6]. However, despite the fact that NSS is preferable from a surgical point of view, patients’ QoL after NSS remains unclear.

The 5-year relative survival rate for stage T1 RCC is around 93% [1]. Thus, oncological outcomes, as well as potential differences between QoL and complication rate after different NSS procedures, are important considerations. In addition, surgical complications present a risk of prolonging recovery [7] and decreasing QoL after NSS [8].

The aim of this study was to identify and summarize results from original studies investigating QoL and complications after NSS due to stage T1 RCC. The objectives were to [1] review the current literature on QoL after NSS and [2] identify differences between NSS procedures with regard to (i) QoL and (ii) complications.

## Methods

### Protocol and registration

This review is registered in PROSPERO (registration number: CRD42020155594). The findings have been reported in accordance with the Preferred Reporting Items for Systematic review and Meta-Analysis (PRISMA) guidelines [9].

### Eligibility criteria

Studies that enrolled adult (>18 years) participants with stage T1 RCC were eligible for inclusion. We restricted eligibility to those studies with a limit of 70% pathologically proven RCC and that provided details on the reported malignancy. The limit of 70% for pathologically proven RCC was established because performing a biopsy in patients with suspected RCC is not routinely carried out prior to treatment worldwide. If a study collected data on tumors larger than stage T1 or enrolled patients with metastatic disease, it was only included in our analysis if the data were stratified by size and/or T-stage.

We included studies that carried out the following types of NSS: PN, CA, RFA, or MWA. In addition, we included different types of procedures, e.g., open, laparoscopic, robot-assisted, or percutaneous/image-guided, with the exception of salvage procedures or procedures following oncological therapy. If a study included other treatment types, such as radical nephrectomy (RN), it was only included if the data were stratified by treatment type. We included studies presenting QoL measures with or without information on complications. We had no restriction on the instruments used to assess QoL or complications.

All study designs, except case reports and retrospective case series, were included. We added no study-age restrictions, but included only studies published in English.

### Information sources and search strategy

In September 2020, we carried out a systematic search of Cinahl, MEDLINE, EMBASE, Scopus, PsycInfo, and the Cochrane Library and repeated the search in September 2021. The search strategy was defined in close cooperation with an information specialist. References from systematic reviews and the studies included in our analysis were manually searched and cross-referenced to ensure completeness. Additionally, PROSPERO was searched for ongoing or recently completed systematic reviews relevant to our criteria. ProQuest Dissertations & Theses Global were searched for grey literature. Search terms were developed according to the PICO framework [10] as shown in Table 1. In addition to medical subject headings, we performed a free-text search using truncation, proximity, and phrase searches. Search strings are listed in [Additional file 1].

**Table 1:** PIO—search terms in MEDLINE

Population Renal cell carcinoma	Intervention Nephron-sparing treatment	Outcome Quality of life
Kidney or renal adj3 cancer* or carcinoma* or neoplasm* or tumo?r* Renal cell carcinoma exp Carcinoma, Renal Cell Kidney Neoplasm exp Kidney Neoplasms Localised renal cell carcinoma Localized renal cell carcinoma Organ sparing treatment exp Organ Sparing Treatments	Nephron sparing treatment Nephron sparing surgery Renal sparing treatment Renal sparing surgery Kidney sparing treatment Kidney sparing surgery Partial nephrectomy Minimal* invasive adj3 procedure Minimal* invasive adj3 treatment Minimal* invasive adj3 surgery Robot* adj3 partial nephrectomy exp Ablation Techniques Thermal ablation exp Cryosurgery Cryoablation Cryo-surgery Cryo-therapy Percutaneous adj3 cryoablation Laparoscopic cryoablation Microwave ablation Radiofrequency ablation Radiofrequency Ablation RFA	Quality of life exp "Quality of Life" exp "Surveys and Questionnaires" QoL Health related quality of life Health-related quality of life HRQoL HR-QoL Quality of life questionnaire* SF-36 Short form 36 SF-12 Short form 12 European Organisation for Research and Treatment of Cancer EORTC EORTC QLQ c-30 EQ-5D EQ5D exp Health Status EuroQoL exp Patient Reported Outcome Measures Patient Reported Outcome Measures PRO Quality of wellbeing Quality of well-being Cancer Rehabilitation Evaluation System-Short form CARES-SF Convalescence and recovery CARE Functional assessment of cancer therapy-general Fact-g Functional assessment of cancer therapy-Kidney Symptom Index FKSI Renal cell carcinoma symptom index RCC-SI

### Screening and study selection

All studies were uploaded to Endnote and managed with Covidence.org (www.covidence.org). Duplicates were removed both in Endnote and again after importation to Covidence. Two independent reviewers completed TiAb screening and full-text screening and performed quality assessment and data extraction. Any disagreement was resolved through discussion.

Customized tables were developed prior to data extraction. The tables were piloted and refined to fit study characteristics and outcomes of interest. Two reviewers independently extracted the data. The following study characteristics and results were extracted: bibliographic information, country of study (based on country of recruited patients), aim, study design (including treatment type and response rate), population (gender, age, time since treatment), outcome instrument for QoL and complications, results, and conclusion. Regarding outcomes, we extracted data on QoL and complications at each time point.

### Quality assessment

Quality assessment was performed using the Critical Appraisal Skills Programme (CASP) checklists for cohort studies and randomized controlled trials [11, 12]. All studies included were assessed independently by two reviewers. Indeterminate criteria fulfilment resulted in a discussion based on the italicized prompts listed under each question in CASP, until consensus was reached. No study was excluded due to a low quality. However, the study quality was taken into consideration in the interpretation of the results and in the conclusions of this review.

### Data synthesis and interpretation

Based on the substantial heterogeneity of the studies included, primarily with regard to clinical diversity, we carried out a narrative synthesis of the data in accordance with the Guidance on the Conduct of Narrative Synthesis in Systematic Reviews [13].

## Results

### Study selection

**Fig. 1:**
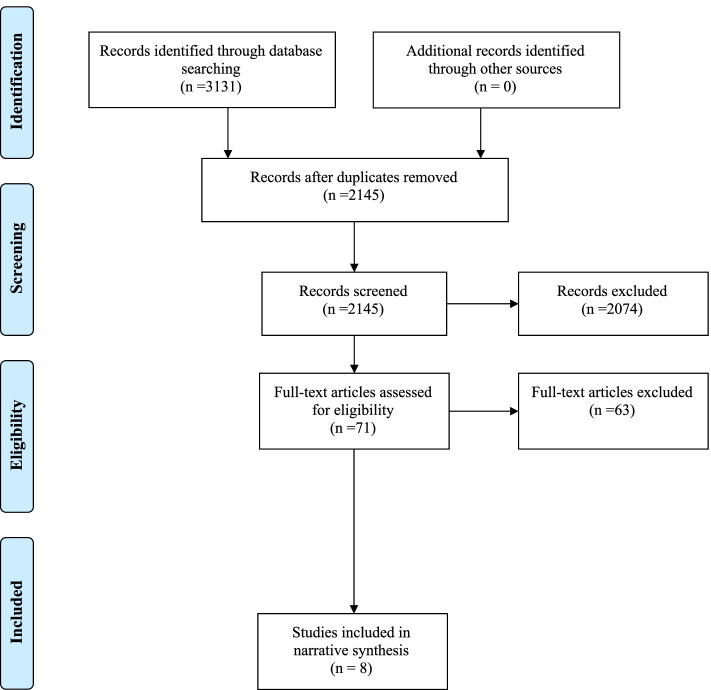
PRISMA flow diagram

After removing duplicates in Endnote and Covidence, 2145 studies were screened against their title and abstract. Overall, 71 studies were included for full-text reading, which resulted in eight studies eligible for inclusion. Two additional studies were identified as relevant when the search was rerun in September 2021. Details are presented in Fig. 1.

### Study characteristics

The eight studies included in the narrative synthesis were published from 2001 to 2021. Four studies were published between 2001 and 2007 [14–17], and four of the most recent studies from 2019 to 2021 [18–21]. Three studies included patients treated from 1985 to 1999 [14–16], and five studies included patients treated from 2004 to 2018 [17–21]. Three studies recruited patients from Japan [15, 17, 21], and one study recruited patients from China [17], USA [13], Italy [15], Netherlands [18], and Canada [20], respectively. All studies included reported QoL measures, and half of the studies included baseline QoL assessments [17, 19–21]. Seven studies reported complications due to treatment [14, 15, 17, 19–21]. Six studies focused on PN [14–16, 18, 20, 21], while the remaining two studies reported ablative therapies, including one percutaneous radiofrequency ablation (RFA) [17], and one both percutaneous cryoablation (PCA) and laparoscopic cryoablation (LCA) [19]. The latter study pooled PCA and LCA into one group, which was labeled as cryoablation (CA) of stage T1 RCC.

QoL outcomes were assessed using a variety of instruments. The validated 36-Item Short Form Health Survey (SF-36) was the most frequently used questionnaire and was used in four of the studies included in this analysis [14, 17–19], and SF-8 was used in one study [21]. Other measurement tools included the Impact of Events Scale (IES) [14], General Health Questionnaire (G.H.Q.) [16], Hospital Anxiety Depression Scale (H.A.D.S) [16], Social Problem Questionnaire (S.P.Q.) [16], Functional Assessment of Cancer Therapy-Kidney Symptom Index-15 (FKSI-15) [19], the European Organisation for Research and Treatment of Cancer Quality-of-life Questionnaire Core 30 (EORTC QlQ C-30) [15], and EQ-5D-5L [20].

Complications were narratively described in four studies [14, 15, 17, 20], and three studies used the Clavien-Dindo classification to assess complications [18, 19, 21]. Only one study explicitly described the time of assessment of complications [19]. Study characteristics are shown in detail in Table 2.

**Table 2:** Summary of study characteristics, key findings, and conclusions of included studies

Study	Aim	Design, treatment, and time of measurement	Population	QoL instrument	Complications	Results	Conclusions
Clark et al. [14] 2001 USA	To analyze the QoL and psychological adjustment after surgical therapy for localized renal cell carcinoma	Retrospective cross-sectional design Treatment period 1990–1997 Elective PN (*n*=30) Mandatory PN (*n*=51) Response rate 75%	74.3% male, 25.7% female mean age 64 years Time since treatment: mean 39 ± 23 months	SF-36, IES, additional questionnaire Distributed via mail.	Self-reported Mean 39 ± 23 months after treatment	The amount of self-reported renal parenchyma remaining was a predictor of several QoL domains. 16.8% reported complicated by problems	QoL is better for patients with more renal parenchyma remaining after surgery for localized renal cell carcinoma.
Shinohara et al. [15] 2001 Japan	To evaluate the impact of PN on postoperative QoL in patients with localized RCC, compared with RN	Retrospective cross-sectional design Treatment period 1986–1996 PN (*n*=12) Response rate 80%	86.7% male, 13.3% female Mean age 61 years Time since treatment: mean 47 ± 40 months	EORTC QLQ-C30 Distributed by mail.	No instrument presented (narrative description) No information of time of assessment.	PN reported higher scores on several QoL domains. 20% complications	Selected patients with localized, small, unilateral RCC, and a normal contralateral kidney will benefit from PN
Ficarra et al. [16] 2002 Italy	To compare the psychological, social well-being and the general state of health in patients who underwent either NSS or RN for T1N0M0 RCC	Retrospective cross-sectional design Treatment period 1985–1999 Elective PN (*n*=56) Response rate not shown	71.4% male, 28.6% female Mean age 58 Time since treatment: mean 62.25 months	G.H.Q., H.A.D.S and S.P.Q Self-administrated during follow-up (physician present).	N.R.	1.8% documented a low level of anxiety 2.3% mild depression 7% impaired general health status 18% documented social problems	Radical surgery seems to eventually cause more negative impact on the psychological well-being than NSS.
Onishi et al. [17] 2007 Japan	To assess the changes in HRQoL during a follow-up period in patients treated with percutaneous RFA or LRN for small RCC	Prospective cohort study Treatment period 2004–2006 Percutaneous RFA (*n*=20) Response rate not shown	75% male, 25% female Mean age 65.9 Time since treatment: 1 week, 4 weeks, 12 weeks, and 24 weeks	SF-36 Distribution type not reported	No instrument presented (narrative description) No information of time of assessment.	No significant difference but a gradual improvement in SF-36 postoperatively. No major surgical or postoperative complications.	If you look exclusively at HRQoL, RFA could be an alternative treatment for selected patients with small RCC.
Wang et al. [18] 2019 China	To evaluate the technical feasibility and outcomes of 2-μm continuous thulium LLPN and conventional LPN in the treatment of patients with SRMs.	Retrospective cross-sectional design Treatment period 2013–2017 LPN (*n*=28) and LLPN (*n*= 30) Response rate 76.3%	LPN: 77.8% male, 22.2% female LLPN: 66.7% male, 33.3% female LPN: mean age 61.2 years LLPN: mean age 63.5 years Time since treatment: 12 months	SF-36 Distribution type not reported	Clavien-Dindo classification system. No information of time of assessment.	No significant differences found in any SF-36 domains between LPN and LLPN. LPN = One intraoperative complication. Postoperative complications Clavien-Dindo grade 1–2: LPN (*n*) = 5/36 LLPN (*n*) = 3/36	LPN and LLPN has acceptable and similar results regarding complications and HRQoL outcome.
Sandbergen et al. [19] 2020 Netherlands	Longitudinal assessment of HRQoL differences in patients with localized renal masses according to treatment strategy.	Prospective cohort study Treatment period 2011–2014 PCA (*n*=11) LCA (*n*=13) Response rate 74.2%	66.7% male, 33.3% female Mean age 70.1 years Time since treatment: 1, 3, and 12 months	SF-36 and FKSI-15 Distributed by mail	Clavien-Dindo classification. Complications within 90 days	Significant difference in social functioning and physical role limitations after one month favoring CA over PN Patients recovered to baseline values on all SF-36 domains 12 months after treatment. 12.5% (*n*=3) complications Clavien-Dindo grade 1 (*n*= 2) Clavien-Dindo grade 2 (*n*=1)	In the short-term HRQoL outcomes favor a minimally invasive approach, but at mid-term these advantages are no longer apparent.
Breau et al. [20] 2021 Canada	To assess the effect of renal hypothermia during OPN on postoperative kidney function. Secondary outcome: Quality of life changes	Prospective, RCT Randomized 1:1 Treatment period 2012–2016 OPN Hypothermia (*n*=92) OPN Controls (*n*=91) Response rate 83.6%	OPN hypothermia: 59% male, 41% female Mean age 58 years OPN controls: 64% male, 36% female Mean age 63 years Time since treatment: 12 months	EQ-5D-5L Distribution type not reported	Adverse effects grouped under labels (ileus, wound infections etc.) Up to 1-year post-operative.	Hypothermia: Mean global health score of 79.3 (baseline) to 82.0 (at 1-year) Controls: Mean global health score of 79.6 (baseline) to 81.2 (at 1-year) No significant change or difference between groups on levels of global health. 10% versus 17% complications (hypothermia versus controls)	OPN did not impact patient reported QoL 12 months after surgery. Nor did renal hypothermia during OPN.
Watanabe et al. [21] 2021 Japan	To investigate the changes in health-related quality of life outcomes in patients with SRM who underwent RAPN	Prospective cohort study Treatment period 2016–2018 RAPN *n*=100 Response rate 100%	64% male, 36% female Mean age 62.6 years Time since treatment: 3, 6 and 12 months	SF-8 Distribution type not reported	Clavien-Dindo classification system. No information of time of assessment.	No individual QoL score were significantly inferior to baseline. BP and RE were significant improved after 3 and 6 months. MH and MCS scores significant improved after 3, 6, and 12 months. 14% complications. Clavien-Dindo grade 1 (*n*= 6) Clavien-Dindo grade 2 (*n*=4) Clavien-Dindo grade 3 (*n*=3) Clavien-Dindo grade 4 (*n*=1)	RAPN shows favorable HRQoL outcomes up to 12 months after surgery. Particularly increasing mental health among patients under the age of 65 years.

*QoL* quality of life, *PN* partial nephrectomy, *SF-36* 36-Item Short Form Health Survey, *IES* the Impact of Events Scale, *NSS* nephron sparing surgery, *EORTC QLQ C30* the European Organisation for Research and Treatment of Cancer Quality-of-life Questionnaire Core 30, *G.H.Q*. General Health Questionnaire, *H.A.D.S* Hospital Anxiety Depression Scale, *S.P.Q*. Social Problem Questionnaire, *RN* radical nephrectomy, *HRQoL* health-related quality of life, *RFA* radiofrequency ablation, *LRN* laparoscopic radical nephrectomy, *LPN* laparoscopic partial nephrectomy, *LLPN* laser-assisted laparoscopic partial nephrectomy, *SRM* small renal masses, *PCA* percutaneous cryoablation, *LCA* laparoscopic cryoablation, *OPN* open partial nephrectomy, *SRM* small renal masses, *RAPN* robot-assisted partial nephrectomy, *BP* bodily pain, *RE* role limitations because of physical health problems, *MH* mental health, *MCS* mental health component summary

### Quality assessment

We adjusted question six of the CASP checklist for cohort studies with regard to follow-up, given that we included four cross-sectional studies, by adding the option of entering “not applicable” (n/a) to the response choices [11]. Overall, we found that all observational studies included in our analysis had a clearly focused objective and recruited patients in an acceptable way. Two studies did not account for possible confounding factors in the study design or analysis [17, 21], and one of them was unclear whether the follow-up of subjects was complete, as no data on response rate or subjects lost to follow-up were presented [17]. We applied “Can’t tell” to five studies with regard to the applicability of the results to the local population [14–17, 21], mainly due to cultural differences and the age of the publication, due to the rapid developments in surgical treatment for RCC and NSS [4]. The only RCT study included received a “yes” to all questions in the CASP Randomized Controlled Trials Checklist [12]. In Table 3, we present details of the quality assessment.

**Table 3:** Quality assessment using a modified version of the CASP checklist for cohort studies [11] and CASP Randomized Controlled Trials Checklist [12]

Study (observational)	Clearly focused question	Recruited subjects in an acceptable way	Exposure accurately measured to minimize bias	Outcome accurately measured to minimize bias	Identified all important confounding factors	Account of the confounding factors in design and/or analysis?	Was the follow up of subjects complete enough?	Was the follow up of subjects long enough?	Do you believe the results?	Can the results be applied to the local population?	Do the results fit with other available evidence?	Implications of this study for practice?
Clark et al. [14]	Yes	Yes	Yes	Yes	Yes	Yes	n/a	n/a	Yes	Can’t tell	Yes	Can’t tell
Shinohara et al. [15]	Yes	Yes	Yes	Yes	Yes	Yes	n/a	n/a	Yes	Can’t tell	Yes	Can’t tell
Ficarra et al. [16]	Yes	Yes	Yes	Yes	Yes	Yes	n/a	n/a	Yes	Can’t tell	Yes	Can’t tell
Onishi et al. [17]	Yes	Yes	Yes	Yes	Yes	No	Can’t tell	Yes	Yes	Can’t tell	Yes	Can’t tell
Wang et al. [18]	Yes	Yes	Yes	Yes	Yes	Yes	n/a	n/a	Yes	Yes	Yes	Yes
Sandbergen et al. [19]	Yes	Yes	Yes	Yes	Yes	Yes	Yes	Yes	Yes	Yes	Yes	Yes
Watanabe et al. [21]	Yes	Yes	Yes	Yes	Yes	Can’t tell	Yes	Yes	Yes	Can’t tell	Yes	Yes
Study (RCT)	Clearly focused question	Randomization to intervention	All patients entering study accounted for	A: Patients “blinded” B: Investigators “blinded” C: “People assessing blinded”	Study groups similar at baseline	Did intervention and control group receive same level of care?	Effects of intervention reported comprehensively	Precision of estimate of treatment effect reported	Benefits of intervention outweigh the harms and costs	Can the results be applied to the local population?	Would the intervention provide greater value to the patients in your care that any of the existing interventions?	
Breau et al. [20]	Yes	Yes	Yes	Yes	Yes	Yes	Yes	Yes	Yes	Yes	Yes	

### Results of individual studies

#### Quality of life

Shinohara et al. and Ficarra et al. found higher scores of QoL after PN compared to RN [15, 16], whereas Clark et al. found no differences in the SF-36 domains between mandatory PN vs. elective PN vs. RN [14]. However, Clark et al. found that self-reported remaining renal parenchyma correlated positively with several QoL domains [14]. In four studies with a longitudinal design, Onishi et al., Sandbergen et al., Breau et al., and Watanabe et al. presented changes over time from baseline measurements [17, 19–21]. Sandbergen et al. reported a small decrease in QoL at one month compared to baseline with regard to the “role-physical functioning and social functioning” after CA regardless of LCA or PCA, whereas Onishi et al. reported no changes in any SF-36 domains 1 week after RFA, compared to baseline. However, they presented figures indicating decreased QoL scores for “bodily pain” and “role-emotional functioning” 1 week after RFA compared to baseline. Furthermore, Onishi et al. report a gradual increase in all SF-36 domains up to 24 weeks after RFA. Watanabe et al. showed similar results regarding all QoL scores after robot-assisted PN (RAPN). Sandbergen et al. found that all patients recovered to baseline QoL values 12 months after PCA and LCA. Likewise, Breau et al. found no significant change in levels of global health 12 months after open PN (OPN). Wang et al. reported no statistically significant differences in any of the SF-36 domains between laser-assisted partial nephrectomy (LLPN) and laparoscopic partial nephrectomy (LPN) 12 months after treatment [18]. A summary of key findings is provided in Table 2.

There was substantial heterogeneity in measurement tools and time-periods of measurement in the included studies. Five out of eight studies used the SF-36 questionnaire, or a subset thereof, to assess QoL [14, 17, 19, 21]. Wang et al., Sandbergen et al., and Watanabe et al. used the SF-36 or SF-8 12 months after treatment [18, 19, 21]. Sandbergen et al. and Watanabe et al. reported their results on graphs, making comparisons difficult [19, 21]. Clark et al. used SF-36 with a follow-up of 39 ± 23 months and do not show results stratified by treatment type [14]. Onishi et al. used SF-36 at 1 week, 1 month, 3 months, and 6 months after treatment and reported graphical results of differences in values from baseline [17]. The two remaining studies included in our analysis used other QoL measurement tools. Therefore, a comparison was not possible.

#### Complications

All studies reported complications after treatment except for one [16]. Sandbergen et al., Wang et al., and Watanabe et al. reported complications that were graded according to the Clavien-Dindo classification [18, 19, 21], and the remaining authors presented narrative descriptions of peri- and/or postoperative complications [14, 15, 17, 20]. Only Sandbergen et al. reported the timing of postoperative complications explicitly within 90 days [19]. Wang et al. reported a minor complication rate of 8.3% after LLPN and 13.9% after LPN, respectively [18], and Watanabe et al. reported a 14% complication rate after RAPN [21]. Shinohara et al. reported a 20% complication rate after OPN, consisting of two patients with minor complications and one patient who required permanent dialysis 5 years postoperatively [15]. Breau et al. reported up to 17% complications after OPN [20], whereas Clark et al., assessed self-reported complications, with 16.8% of their respondents reporting complications and 83.2% reporting no major complications [14]. The two studies on ablative therapies, including percutaneous RFA [17], LCA, and PCA [19], reported no major surgical or postoperative complications, but found a minor complication rate of 12.5% grade 1–2 complications, based on the Clavien-Dindo classification, following PCA and LCA.

#### Synthesis of results

In the eight studies that assessed QoL outcomes after NSS of stage T1 RCC, a total of 491 patients received PN, 24 patients received CA, and 20 patients received RFA. The seven studies that assessed post-treatment complications included 435 patients who were assessed after PN, 24 patients after CA, and 20 patients after RFA. Comparison of QoL outcomes across the eight studies was not possible due to the lack of exact QoL results presented in the individual studies, and the lack of separate data for NSS. QoL results were descriptively reported or reported on graphs, which did not allow for data extraction for comparison or meta-analysis. In half of the studies regarding PN (*n*= 150), PN was compared to RN in the original studies and in a retrospective design. Across these studies, we found higher, post-treatment scores of QoL after PN compared to RN. In the prospective studies of PN, we found that QoL increased or returned to baseline levels. No studies were identified that compared PN to ablative therapies for stage T1 RCC. One study showed a small decrease in QoL at the short-term follow-up after CA, but found that patients returned to baseline levels of QoL at the mid-term follow-up. With regard to RFA, one study reported no decrease in QoL after treatment and a gradual increase during a follow-up of 24 weeks. Our analysis of the eight included studies found a complication rate up to 20% after PN, up to 12.5% after CA, and no complications reported after RFA.

## Discussion

### Summary of evidence

The aim of this systematic review was to investigate QoL after NSS and identify differences between NSS procedures regarding QoL and complications. We only identified eight studies, all of which had relatively small patient populations. The included studies were heterogenous with regard to patients, outcome measurement tools, and study design. The four studies with a baseline QoL reported either a trend towards normalizing to baseline QoL after 12 months [19, 20] or a gradual increase in QoL up to 12 months after treatment [17, 21].

A systematic review by MacLennan et al. from 2012 found a paucity of QoL outcomes following surgical management of localized RCC [6]. Even though, in this review, we included four studies published after 2012 [18–21] and included percutaneous procedures and retrospective studies, our findings support the findings of MacLennan et al. [6]. Research on QoL after NSS is sparse. Rossi et al. evaluated the evidence regarding QoL following different management strategies for localized RCC and recommended the need for validated and reproducible QoL measurement tools [22]. We focused on NSS and, contrary to the previous literature review, we conducted a systematic search six databases in close cooperation with an information specialist. Half of the studies included in this review were not included in the literature review by Rossi et al. [18–21]. Nonetheless, our findings support those of Rossi et al. There is still a need, however, for further research addressing QoL after NSS with the use of validated measurement tools and, preferably, a solid study design.

The heterogeneity of the studies included in our review compromised our aim to place our results in context with previous research. Nevertheless, our findings suggest that PN is superior, to some extent, to RN with regard to QoL following treatment of tumors stage T1 [14, 16]. Similar results were found in studies that included patients with more advanced tumors, by Poulakis et al. [23] and Azawi et al. [8]. Even though Poulakis et al. did not find major differences in QoL between RN and PN overall, the authors found a significant difference in several QoL domains between RN and elective PN [23]. Furthermore, Poulakis et al. found that tumor size was significantly associated with a return to baseline QoL, which is corroborated by the results of this review. The findings by Poulakis et al. enhanced the decision to focus this review on stage T1 tumors. In addition, it highlights the relevance of reporting tumor size when reporting QoL outcomes.

The variety of QoL measurement tools and the heterogeneous study designs contributed to the challenges of evaluating QoL after different NSS procedures. Five studies used the generic QoL measurement tool SF-36, or a subset thereof (SF-8), which was not designed to specifically address QoL in connection with cancer treatment. However, SF-36 is the most commonly used QoL assessment tool and thus enables the comparison of results across studies. SF-36 is designed for a 4-week recall period or an acute form with a 1 week recall period. However, one study used the SF-36 after 1 week, without specifying which questionnaire was used, weakening both the external and internal validity of that study.

We excluded studies that did not address pathology or included less than 70% pathologically verified RCC. This might contribute to the low number of studies using ablative therapies. Biopsy is not routinely performed prior to ablative therapies, creating a risk of nondiagnostic results [24, 25]. We argue that QoL could be influenced by whether or not patients have a benign or malignant tumor. Novara et al. found that patients with benign tumors had significantly better scores when it came to role limitation due to emotional problems 12 months after surgical treatment of RCC [26]. In contrast to Novara et al., Beemster et al. reported that patients treated with LCA due to malignant tumors had higher scores on general health perception than patients with benign tumors [27]. This could reflect a scenario in which patients feel relieved after a curative treatment for a malignant tumor, whereas patients with benign tumors have undergone a treatment without having had cancer. Beemster et al. did not investigate fear of recurrence, which might be a greater concern for patients with malignant tumors. In addition, the study population was relatively small (*n*=57) [27]. Poulakis et al. found that fear of recurrence correlated with tumor size and that patients who underwent a mandatory PN had a higher degree of concern compared to those who’d undergone elective PN and RN [23]. Regardless of whether malignancy leads to decreased or increased QoL after treatment of RCC, it appears to potentially influence QoL outcomes, making it relevant to add pathology to the inclusion criteria of this review. It is evident that the patient population for the different types of NSS varied across the eight studies included. Other researchers have experienced this challenge. Consequently, Shinohara et al. changed the selection criteria for PN halfway through their inclusion period [15]. Almost half of the studies included in this review recruited patients before ablative therapies were introduced as a potential treatment for RCC [14–16]. Furthermore, ablation therapies are primarily offered to elderly patients unfit for surgery [5]. Sandbergen et al. only included clinical T1a tumors for CA [19], and in the study by Onishi et al., patients receiving RFA were significantly older than patients receiving PN [17]. Also, the fact that half of the studies included in our review are retrospective [14–16, 18] represents a weakness in the evidence base of QoL after NSS of stage T1 RCC. In this review, we investigated active types of treatment, but in terms of cancer-specific survival, it has been suggested that active surveillance (AS) could be introduced as a treatment option [28, 29]. However, Alam et al. showed that AS patients had lower QoL compared to those who’d had PN and ablation, likely due to lower baseline health status [28]. Likewise, Goldberg et al. found that patients in AS in a large (*n*=477) Canadian cohort had similar psychological distress compared to patients after surgery and ablation [30].

We found that seven of the eight studies assessed complications. However, the timing and manner of the assessments varied widely. Only three studies reported complications according to an acknowledged classification tool [18, 19, 21], all using the Clavien-Dindo classification [31]: thus, a comparison of complications across studies was not possible. Furthermore, our results on complications are only based on studies that also reported QoL outcomes. However, we found some indication that PN was related to a higher risk of complications compared to ablative therapies. Rivero et al. reported a similar complication rate in a systematic review and meta-analysis from 2018 that compared ablation (*n*=940), CA, and RFA, to PN (*n*=1040) [32] and found a complication rate of 13% versus 17.6% after ablative therapy and PN, respectively [32]. Nevertheless, our findings, based on the seven studies that assessed complications, could also be associated with the number of open procedures in the studies that included PN [14–16, 20]. This could be associated with the fact that most of the studies of PN are dated prior to the standard use of minimally invasive procedures, such as LPN or RAPN [33, 34]. However, our findings on QoL could also be anecdotal due to the limited number of studies included in this review and their relatively small population sizes. Thus, we recommend caution in comparing the results across the studies included.

Gratzke et al. investigated QoL after OPN of stage T1–T3 tumors and showed that patients with a higher complication rate had lower self-reported QoL after surgery [35]. Sandbergen et al. also found a higher complication rate following PN of stage T1–T2 tumors compared to CA, reflecting a decrease in QoL after 1 month [19]. On that basis, the rate of complications is worth measuring when considering QoL outcomes.

Half of the studies included in this review are recent publications, which reflects an increased focus on the value of QoL as an important outcome following NSS. However, the heterogeneity in reporting on QoL outcomes poses a challenge because it prevents us from drawing conclusions to offer suggestions for changes in practice. To our knowledge, RCC-specific QoL instruments are not available, which would explain the diversity of assessments use in the studies included in our review. QoL outcomes should be assessed with validated measurement tools in protocol-driven studies to allow comparative assessment, as suggested by Abu-Ghanem et al. [36]. Likewise, registration of complications should be classified with the use of standardized assessments.

### Limitations

The limited number of studies, as well as the age of half of the studies included, is a limitation of this systematic review. Four out of eight studies were conducted in 2007 or earlier, which limits the relevance of applying the results to current clinical practice. The heterogeneity of the studies precluded a meta-analysis, and the high number of retrospective studies complicated the ability to provide precise answers to the objectives of this review. The inclusion of articles only published in English could be a limitation, whereas one of the strengths of the study was the systematic and thorough search of six databases, and was not limited to study design or the time period in which the study was conducted. In addition, the strict inclusion criteria could be considered a strength of this review, in that patients with heterogenous tumors and disease stages, who were excluded, would not affect the outcome.

## Conclusions

Little evidence is available about QoL following NSS of stage T1 RCC. Half of the studies in this review were retrospective and relatively old. Based on the results of this review, NSS appears to be either superior, or comparable, to other treatment alternatives with regard to QoL outcomes. Additionally, based on the studies included in this systematic review, it appears that PN is associated with a higher complication rate compared to that of ablation therapies. Further research within the field of QoL and complications after NSS of pathologically verified stage T1 RCC is highly recommended, preferably with larger cohorts, validated tools, and rigorous study designs.

Title of data: The search strategies applied to the six databases

Description of data: Additional file 1 includes search strategies and terms applied in Cinahl, Medline, Embase, Scopus, PsykInfo and Cochrane Library

## Supplementary Information

**Additional file 1.**

## Data Availability

All data analyzed in this study are included in this published article and supplementary.

## Abbreviations

RCC: Renal cell carcinoma
PN: Partial nephrectomy
RFA: Radiofrequency ablation
CA: Cryoablation
MWA: Microwave ablation
NSS: Nephron-sparing surgery
QoL: Quality of life
RN: Radical nephrectomy
PRISMA: Preferred Reporting Items for Systematic review and Meta-Analysis
TJ: Theresa Junker
LD: Louise Duus
BN: Birgitte Nørgaard
CASP: Critical Appraisal Skills Programme
PCA: Percutaneous cryoablation
LCA: Laparoscopic cryoablation
SF-36: 36-Item Short Form Health Survey
IES: Impact of Events Scale
G.H.Q.: General Health Questionnaire
H.A.D.S: Hospital Anxiety Depression Scale
S.P.Q: Social Problem Questionnaire
FKSI-15: Functional Assessment of Cancer Therapy-Kidney Symptom Index-15
EORTC QlQ C-30: European Organisation for Research and Treatment of Cancer Quality-of-life Questionnaire Core 30
n/a: Not applicable
RAPN: Robot-assisted partial nephrectomy
OPN: Open partial nephrectomy
LLPN: Laser-assisted partial nephrectomy
LPN: Laparoscopic partial nephrectomy
BSBR: Benjamin S.B. Rasmussen
NA: Nessn Azawi
LL: Lars Lund
OG: Ole Graumann
