# Supplementary material for: Quality of life and complications after nephron-sparing treatment of renal cell carcinoma stage T1—a systematic review
Source: Syst Rev. 2022 Jan 4;11:4. doi: 10.1186/s13643-021-01868-2 (PMC8725354; doi:10.1186/s13643-021-01868-2)
Supplement: Supplementary file 1 — Additional file 1. [file 13643_2021_1868_MOESM1_ESM.docx]

**Quality of life and complications after nephron sparing treatment of renal cell carcinoma stage T1 - A systematic review**

**Additional file 1**

**The** **search strategies applied to the six databases**

**Cinahl**

September 2020 n= 228

September 2021 n= 250

| S1 | (Kidney OR renal) n2 (cancer* OR carcinoma* OR neoplasm* OR tumo?r*) |
| --- | --- |
| S2 | (MH "Kidney Neoplasms+") |
| S3 | ((locali?ed) n2 (renal OR kidney) n2 (carcinoma*)) |
| S4 | S1 OR S2 OR S3 |
| S5 | "Nephron sparing treatment" |
| S6 | Renal sparing treatment |
| S7 | "Renal sparing surgery" |
| S8 | "Nephron sparing surgery" |
| S9 | "Kidney sparing treatment" |
| S10 | "Kidney sparing surgery" |
| S11 | "Partial nephrectomy" |
| S12 | (MH "Minimally Invasive Procedures+") OR "Minimal* invasive n2 procedure" |
| S13 | "Minimal* invasive n2 treatment" |
| S14 | "Minimal* invasive n2 surgery" |
| S15 | "Laparoscopic partial nephrectomy" |
| S16 | "Laparoscop* partial nephrectomy" |
| S17 | (MH "Robotic Surgical Procedures") OR "Robot-assisted partial nephrectomy" |
| S18 | "Robotic assisted partial nephrectomy" |
| S19 | "Robot* n2 partial nephrectomy" |
| S20 | (MH "Ablation Techniques+") OR (MH "Radiofrequency Ablation") OR "Thermal ablation" |
| S21 | (MH "Ablation Techniques+") OR "Ablation Techniques" |
| S22 | (MH "Cryosurgery") OR (MH "Cryotherapy") OR "Cryoablation" |
| S23 | Cryosurgery |
| S24 | "Cryo-surgery" |
| S25 | "Cryo-therapy" |
| S26 | "Percutaneous n2 cryoablation" |
| S27 | "Laparoscopic cryoablation" |
| S28 | "Microwave ablation" |
| S29 | (MH "Radiofrequency Therapy+") OR "Radiofrequency ablation" |
| S30 | "RFA" |
| S31 | "mwa" |
| S32 | ""organ sparing treatment"" |
| S33 | ""organ sparing surgery"" |
| S34 | S5 OR S6 OR S7 OR S8 OR S9 OR S10 OR S11 OR S12 OR S13 OR S14 OR S15 OR S16 OR S17 OR S18 OR S19 OR S20 OR S21 OR S22 OR S23 OR S24 OR S25 OR 2S6 OR S27 OR S28 OR S29 OR S30 OR S31 OR S32 OR S33 |
| S35 | (MH "Quality of Life+") OR "Quality of life" OR (MH "Quality of Life (Iowa NOC)") OR (MH "Well-Being (Iowa NOC)") |
| S36 | "QoL" |
| S37 | (MH "Structured Questionnaires") OR (MH "Surveys+") OR (MH "Short Form-36 Health Survey (SF-36)") OR (MH "Survey Research") OR (MH "OARS Multidimensional Functional Assessment Questionnaire") OR (MH "Health Perceptions Questionnaire") OR "Surveys and Questionnaires" OR (MH "Personal Resource Questionnaire") |
| S38 | (MH "Health and Life Quality (Iowa NOC)+") OR "Health related quality of life" |
| S39 | "Health-related quality of life" |
| S40 | "HRQoL" |
| S41 | "HR-QoL" |
| S42 | "Quality of life questionnaire*" |
| S43 | (MH "Short Form-36 Health Survey (SF-36)") OR "SF-36" |
| S44 | (MH "Short Form-36 Health Survey (SF-36)") OR "Short form 36" |
| S45 | "SF-12" |
| S46 | "Short form 12" |
| S47 | "European Organisation for Research and Treatment of Cancer" |
| S48 | "EORTC" |
| S49 | "EORTC QlQ c-30" |
| S50 | "EQ5D" |
| S51 | "EQ-5D" |
| S52 | "EuroQoL" |
| S53 | (MH "Health Status+") OR "Health Status" |
| S54 | (MH "Patient-Reported Outcomes") OR "Patient Reported Outcome Measures" |
| S55 | "Patient Reported Outcome Measures" OR (MH "Patient-Reported Outcomes") |
| S56 | "PRO" |
| S57 | "Quality of wellbeing" |
| S58 | "quality of well-being" |
| S59 | "Cancer Rehabilitaion Evaluation System-Short form" |
| S60 | "CARES-SF" |
| S61 | "CARE" |
| S62 | "Functional assessment of cancer therapy-general" |
| S63 | "Fact-g" |
| S64 | Functional assessment of cancer therapy-Kidney Symptom Index |
| S65 | "rcc-si" OR "FKSI" OR "renal cell carcinoma symptom index" |
| S66 | S36 OR S37 OR S38 OR S39 OR S40 OR S41 OR S42 OR S43 OR S44 OR S45 OR S46 OR S47 OR S48 OR S49 OR S50 V S51 OR S52 OR S53 OR S54 OR S55 OR S56 OR S57 OR S58 OR S59 OR S60 OR S61 OR S62 OR S63 OR S64 OR S65 |
| S67 | S4 AND S34 AND S66 |

**Medline**

September 2020 n= 981

September 2021 n= 1013

| 68 | 65 and 66 and 67 |
| --- | --- |
| 67 | 59 or 60 or 61 or 62 or 63 |
| 66 | 24 or 25 or 26 or 27 or 28 or 29 or 30 or 31 or 32 or 33 or 34 or 35 or 36 or 37 or 38 or 39 or 40 or 41 or 42 or 43 or 44 or 45 or 46 or 47 or 48 or 49 or 50 or 51 or 52 or 53 or 54 or 55 or 56 or 57 or 58 |
| 65 | 1 or 2 or 3 or 4 or 5 or 6 or 7 or 8 or 9 or 10 or 11 or 12 or 13 or 14 or 15 or 16 or 17 or 18 or 19 or 20 or 21 or 22 or 23 or 64 |
| 64 | organ sparing treatment.mp. or exp Organ Sparing Treatments/ |
| 63 | localized renal cell carcinoma.mp. |
| 62 | localised renal cell carcinoma.mp. |
| 61 | Kidney Neoplasm.mp. or exp Kidney Neoplasms/ |
| 60 | Renal cell carcinoma.mp. or exp Carcinoma, Renal Cell/ |
| 59 | ((Kidney or renal) adj3 (cancer* or carcinoma* or neoplasm* or tumo?r*)).mp. [mp=title, abstract, original title, name of substance word, subject heading word, floating sub-heading word, keyword heading word, organism supplementary concept word, protocol supplementary concept word, rare disease supplementary concept word, unique identifier, synonyms] |
| 58 | RCC-SI.mp. |
| 57 | renal cell carcinoma symptom index.mp. |
| 56 | FKSI.mp. |
| 55 | Functional assessment of cancer therapy-Kidney Symptom Index.mp. |
| 54 | Fact-g.mp. |
| 53 | Functional assessment of cancer therapy-general.mp. |
| 52 | CARE.mp. |
| 51 | (Convalescence and recovery evaluation).mp. [mp=title, abstract, original title, name of substance word, subject heading word, floating sub-heading word, keyword heading word, organism supplementary concept word, protocol supplementary concept word, rare disease supplementary concept word, unique identifier, synonyms] |
| 50 | CARES-SF.mp. |
| 49 | Cancer Rehabilitation Evaluation System-Short form.mp. |
| 48 | Quality of well-being.mp. |
| 47 | Quality of wellbeing.mp. |
| 46 | pro.mp. |
| 45 | Patient Reported Outcome Measures.mp. |
| 44 | exp Patient Reported Outcome Measures/ |
| 43 | EuroQoL.mp. |
| 42 | exp Health Status/ |
| 41 | EQ5D.mp. |
| 40 | EQ-5D.mp. |
| 39 | EORTC QlQ c-30.mp. |
| 38 | EORTC.mp. |
| 37 | (European Organisation for Research and Treatment of Cancer).mp. [mp=title, abstract, original title, name of substance word, subject heading word, floating sub-heading word, keyword heading word, organism supplementary concept word, protocol supplementary concept word, rare disease supplementary concept word, unique identifier, synonyms] |
| 36 | Short form 12.mp. |
| 35 | SF-12.mp. |
| 34 | Short form 36.mp. |
| 33 | Short form 36.mp. |
| 32 | SF-36.mp. |
| 31 | Quality of life questionnaire*.mp. |
| 30 | HR-QoL.mp. |
| 29 | HRQoL.mp. |
| 28 | Health-related quality of life.mp. |
| 27 | Health related quality of life.mp. |
| 26 | QoL.mp. |
| 25 | exp "Surveys and Questionnaires"/ |
| 24 | Quality of life.mp. or exp "Quality of Life"/ |
| 23 | RFA.mp. |
| 22 | exp Radiofrequency Ablation/ |
| 21 | Radiofrequency ablation.mp. |
| 20 | Microwave ablation.mp. |
| 19 | Laparoscopic cryoablation.mp. |
| 18 | (Percutaneous adj3 cryoablation).mp. [mp=title, abstract, original title, name of substance word, subject heading word, floating sub-heading word, keyword heading word, organism supplementary concept word, protocol supplementary concept word, rare disease supplementary concept word, unique identifier, synonyms] |
| 17 | Cryo-therapy.mp. |
| 16 | Cryo-surgery.mp. |
| 15 | Cryoablation.mp. |
| 14 | exp Cryosurgery/ |
| 13 | Thermal ablation.mp. |
| 12 | exp Ablation Techniques/ |
| 11 | (Robot* adj3 partial nephrectomy).mp. [mp=title, abstract, original title, name of substance word, subject heading word, floating sub-heading word, keyword heading word, organism supplementary concept word, protocol supplementary concept word, rare disease supplementary concept word, unique identifier, synonyms] |
| 10 | (Minimal* invasive adj3 surgery).mp. [mp=title, abstract, original title, name of substance word, subject heading word, floating sub-heading word, keyword heading word, organism supplementary concept word, protocol supplementary concept word, rare disease supplementary concept word, unique identifier, synonyms] |
| 9 | (Minimal* invasive adj3 treatment).mp. [mp=title, abstract, original title, name of substance word, subject heading word, floating sub-heading word, keyword heading word, organism supplementary concept word, protocol supplementary concept word, rare disease supplementary concept word, unique identifier, synonyms] |
| 8 | (Minimal* invasive adj3 procedure).mp. [mp=title, abstract, original title, name of substance word, subject heading word, floating sub-heading word, keyword heading word, organism supplementary concept word, protocol supplementary concept word, rare disease supplementary concept word, unique identifier, synonyms] |
| 7 | Partial nephrectomy.mp. |
| 6 | Kidney sparing surgery.mp. |
| 5 | Kidney sparing treatment.mp. |
| 4 | Renal sparing surgery.mp. |
| 3 | Renal sparing treatment.mp. |
| 2 | Nephron sparing surgery.mp. |
| 1 | Nephron sparing treatment.mp. |

**Embase**

September 2020 n= 695

September 2021 n= 738

| 1 ((Kidney or renal) adj3 (cancer* or carcinoma* or neoplasm* or tumo?r*)).mp. [mp=title, abstract, heading word, drug trade name, original title, device manufacturer, drug manufacturer, device trade name, keyword, floating subheading word, candidate term word] |
| --- |
| 2 exp kidney tumor/ or exp kidney carcinoma/ or exp renal cell carcinoma/ |
| 3 exp kidney tumor/ |
| 4 exp renal cell carcinoma/ |
| 5 exp kidney cancer/ |
| 6 localised renal cell carcinoma.mp. |
| 7 localized renal cell carcinoma.mp. |
| 8 Nephron sparing treatment.mp. |
| 9 exp nephron sparing surgery/ |
| 10 Nephron sparing surgery.mp. |
| 11 Renal sparing treatment.mp. |
| 12 Renal sparing surgery.mp. |
| 13 Kidney sparing treatment.mp. |
| 14 Kidney sparing surgery.mp. |
| 15 exp partial nephrectomy/ |
| 16 Partial nephrectomy.mp. |
| 17 (Minimal* invasive adj3 procedure).mp. [mp=title, abstract, heading word, drug trade name, original title, device manufacturer, drug manufacturer, device trade name, keyword, floating subheading word, candidate term word] |
| 18 (Minimal* invasive adj3 treatment).mp. [mp=title, abstract, heading word, drug trade name, original title, device manufacturer, drug manufacturer, device trade name, keyword, floating subheading word, candidate term word] |
| 19 (Minimal* invasive adj3 surgery).mp. [mp=title, abstract, heading word, drug trade name, original title, device manufacturer, drug manufacturer, device trade name, keyword, floating subheading word, candidate term word] |
| 20 exp laparoscopic surgery/ |
| 21 (Robot* adj3 partial nephrectomy).mp. [mp=title, abstract, heading word, drug trade name, original title, device manufacturer, drug manufacturer, device trade name, keyword, floating subheading word, candidate term word] |
| 22 Thermal ablation.mp. |
| 23 exp ablation therapy/ |
| 24 Ablation Techniques.mp. |
| 25 exp cryoablation/ |
| 26 Cryoablation.mp. |
| 27 exp cryosurgery/ |
| 28 Cryo-surgery.mp. |
| 29 Cryosurgery.mp. |
| 30 Cryo-therapy.mp. |
| 31 (Percutaneous adj3 cryoablation).mp. [mp=title, abstract, heading word, drug trade name, original title, device manufacturer, drug manufacturer, device trade name, keyword, floating subheading word, candidate term word] |
| 32 Laparoscopic cryoablation.mp. |
| 33 exp microwave thermotherapy/ |
| 34 Microwave ablation.mp. |
| 35 exp radiofrequency ablation/ |
| 36 Radiofrequency ablation.mp. |
| 37 rfa.mp. |
| 38 exp "quality of life"/ |
| 39 Quality of life.mp. |
| 40 exp questionnaire/ |
| 41 QoL.mp. 74675 |
| 42 exp "Surveys and Questionnaires"/ |
| 43 Health related quality of life.mp. |
| 44 Health-related quality of life.mp. |
| 45 HRQoL.mp. |
| 46 HR-QoL.mp. |
| 47 Quality of life questionnaire*.mp. |
| 48 exp Short Form 36/ |
| 49 SF-36.mp. |
| 50 Short form 36.mp. |
| 51 exp Short Form 12/ |
| 52 SF-12.mp. |
| 53 Short form 12.mp. |
| 54 (European Organisation for Research and Treatment of Cancer).mp. [mp=title, abstract, heading word, drug trade name, original title, device manufacturer, drug manufacturer, device trade name, keyword, floating subheading word, candidate term word] |
| 55 EORTC.mp. |
| 56 EORTC QlQ c-30.mp. |
| 57 EQ-5D.mp. |
| 58 EQ5D.mp. |
| 59 EuroQoL.mp. |
| 60 exp health status/ |
| 61 exp patient- reported outcome/ |
| 62 patient-reported outcome.mp. |
| 63 patientreported outcome.mp. |
| 64 Patient Reported Outcome Measures.mp. |
| 65 PRO.mp. |
| 66 exp wellbeing/ |
| 67 Quality of wellbeing.mp. |
| 68 Quality of well- being.mp. |
| 69 CARES-SF.mp. |
| 70 (Convalescence and recovery evaluation).mp. [mp=title, abstract, heading word, drug trade name, original title, device manufacturer, drug manufacturer, device trade name, keyword, floating subheading word, candidate term word] |
| 71 exp health care survey/ or exp urological care/ |
| 72 Functional assessment of cancer therapy- general.mp. |
| 73 Fact-g.mp. |
| 74 Functional assessment of cancer therapy- Kidney Symptom Index.mp. |
| 75 FKSI.mp. |
| 76 renal cell carcinoma symptom index.mp. |
| 77 RCC-SI.mp. |
| 78 organ sparing treatment.mp. |
| 79 organ sparing surgery.mp. |
| 80 1 or 2 or 3 or 4 or 5 or 6 or 7 |
| 81 8 or 9 or 10 or 11 or 12 or 13 or 14 or 15 or 16 or 17 or 18 or 19 or 20 or 21 or 22 or 23 or 24 or 25 or 26 or 27 or 28 or 29 or 30 or 31 or 32 or 33 or 34 or 35 or 36 or 37 or 78 or 79 |
| 82 38 or 39 or 40 or 41 or 42 or 43 or 44 or 45 or 46 or 47 or 48 or 49 or 50 or 51 or 52 or 53 or 54 or 55 or 56 or 57 or 58 or 59 or 60 or 61 or 62 or 63 or 64 or 65 or 66 or 67 or 68 or 69 or 70 or 71 or 72 or 73 or 74 or 75 or 76 or 77 |
| 83 80 and 81 and 82 |

**Scopus**

September 2020 n= 951

September 2021 n= 1056

| #1 | ( ( kidney  OR  renal )  W/2  ( cancer*  OR  carcinoma*  OR  neoplasm*  OR  tumo?r* ) )  OR  ( "localised renal cell carcinoma" )  OR  ( "localized renal cell carcinoma" ) |
| --- | --- |
| #2 | ( ( TITLE-ABS-KEY ( "Nephron sparing treatment" ) )  OR  ( TITLE-ABS-KEY ( "Nephron sparing surgery" ) )  OR  ( TITLE-ABS-KEY ( "renal sparing surgery" ) )  OR  ( TITLE-ABS-KEY ( "renal sparing treatment" ) )  OR  ( TITLE-ABS-KEY ( "kidney sparing treatment" ) )  OR  ( TITLE-ABS-KEY ( "kidney sparing surgery" ) )  OR  ( TITLE-ABS-KEY ( partial  W/2  nephrectomy ) )  OR  ( TITLE-ABS-KEY ( minimal*  W/0  invasive  W/2  procedure ) )  OR  ( TITLE-ABS-KEY ( minimal*  W/0  invasive  W/2  treatment ) )  OR  ( TITLE-ABS-KEY ( organ  W/0  sparing  W/2  treatment ) )  OR  ( TITLE-ABS-K EY ( organ  W/0  sparing  W/2  surgery ) )  OR  ( TITLE-ABS-KEY ( "Ablation Technique*" ) )  OR  ( TITLE-ABS-KEY ( "thermal ablation*" ) )  OR  ( TITLE-ABS-KEY ( "cryosurgery" ) )  OR  ( TITLE-ABS-KEY ( "cryoablation" ) )  OR  ( TITLE-ABS-KEY ( "cryotherapy" ) )  OR  ( TITLE-ABS-KEY ( percutaneous  W/2  cryoablation ) )  OR  ( TITLE-ABS-KEY ( "microwave ablation" ) )  OR  ( TITLE-ABS-KEY ( "Radiofrequency ablation" ) )  OR  ( TITLE-ABS-KEY ( "RFA" ) )  OR  ( TITLE-ABS-KEY ( "MWA" ) ) )  OR  ( "laparoscopic surgery" )  OR  ( "ablation therapy" )  OR  ( "Laparoscopic cryoablation" ) |
| #3 | ( ( TITLE-ABS-KEY ( "renal cell carcinoma symptom index" ) )  OR  ( TITLE-ABS-KEY ( "rcc-si" ) )  OR  ( ( TITLE-ABS-KEY ( "Quality of life" ) )  OR  ( TITLE-ABS-KEY ( "Surveys and Questionnaires" ) )  OR  ( TITLE-ABS-KEY ( "QoL" ) )  OR  ( TITLE-ABS-KEY ( "health related quality of life" ) )  OR  ( TITLE-ABS-KEY ( "HRQOL" ) )  OR  ( TITLE-ABS-KEY ( "Quality of life questionnaire*" ) )  OR  ( TITLE-ABS-KEY ( "SF-36" ) )  OR  ( TITLE-ABS-KEY ( "SF-12" ) )  OR  ( TITLE-ABS-KEY ( "Short form 12" ) )  OR  ( TITLE-ABS-KEY ( "Short form 36" ) )  OR  ( TITLE-ABS-KEY ( "European Organisation for Research and Treatment of Cancer" ) )  OR  ( TITLE-ABS-KEY ( "EORTC" ) )  OR  ( TITLE-ABS-KEY ( "EORTC QlQ C-30" ) )  OR  ( TITLE-ABS-KEY ( "EQ5D" ) )  OR  ( TITLE-ABS-KEY ( "Health Status" ) )  OR  ( TITLE-ABS-KEY ( "euroqol" ) )  OR  ( TITLE-ABS-KEY ( "Patient Reported Outcome Measures" ) )  OR  ( TITLE-ABS-KEY ( "Patient Reported Outcome" ) )  OR  ( TITLE-ABS-KEY ( "PRO" ) )  OR  ( TITLE-ABS-KEY ( "Quality of wellbeing" ) )  OR  ( TITLE-ABS-KEY ( "Quality of well-being" ) )  OR  ( TITLE-ABS-KEY ( "Cancer Rehabilitation Evaluation System-Short form" ) )  OR  ( TITLE-ABS-KEY ( "CARES-SF" ) )  OR  ( TITLE-ABS-KEY ( convalescence  W/2  recovery  W/0  evaluation ) )  OR  ( TITLE-ABS-KEY ( "Functional assessment of cancer therapy-general" ) )  OR  ( TITLE-ABS-KEY ( "Fact-g" ) )  OR  ( TITLE-ABS-KEY ( "Functional assessment of cancer therapy-Kidney Symptom Index" ) )  OR  ( TITLE-ABS-KEY ( "FKSI" ) ) ) )  OR  ( "health care survey" )  OR  ( "urological care" )  OR  ( "patient-reported outcome" )  OR  ( "EQ-5D" )  OR  ( "HR-QoL" )  OR  ( "Health-related quality of life" ) |
| #4 | #1 AND #2 AND #3 |

**PsykInfo**

September 2020 n= 14

September 2021 n= 17

| 1 | Nephron sparing treatment.mp. [mp=title, abstract, heading word, table of contents, key concepts, original title, tests & measures, mesh] |
| --- | --- |
| 2 | Nephron sparing surgery.mp. [mp=title, abstract, heading word, table of contents, key concepts, original title, tests & measures, mesh] |
| 3 | Renal sparing surgery.mp. [mp=title, abstract, heading word, table of contents, key concepts, original title, tests & measures, mesh] |
| 4 | Renal sparing surgery.mp. [mp=title, abstract, heading word, table of contents, key concepts, original title, tests & measures, mesh] |
| 5 | kidney sparing treatment.mp. [mp=title, abstract, heading word, table of contents, key concepts, original title, tests & measures, mesh] |
| 6 | kidney sparing surgery.mp. [mp=title, abstract, heading word, table of contents, key concepts, original title, tests & measures, mesh] |
| 7 | partiel nephrectomy.mp. [mp=title, abstract, heading word, table of contents, key concepts, original title, tests & measures, mesh] |
| 8 | (Minimal* invasive adj3 procedure).mp. [mp=title, abstract, heading word, table of contents, key concepts, original title, tests & measures, mesh] |
| 9 | (Minimal* invasive adj3 treatment).mp. [mp=title, abstract, heading word, table of contents, key concepts, original title, tests & measures, mesh] |
| 10 | (Minimal* invasive adj3 surgery).mp. [mp=title, abstract, heading word, table of contents, key concepts, original title, tests & measures, mesh] |
| 11 | Ablation Techniques.mp. [mp=title, abstract, heading word, table of contents, key concepts, original title, tests & measures, mesh] |
| 12 | Thermal ablation.mp. [mp=title, abstract, heading word, table of contents, key concepts, original title, tests & measures, mesh] |
| 13 | Cryosurgery.mp. [mp=title, abstract, heading word, table of contents, key concepts, original title, tests & measures, mesh] |
| 14 | Cryoablation.mp. [mp=title, abstract, heading word, table of contents, key concepts, original title, tests & measures, mesh] |
| 15 | cryo-surgery.mp. [mp=title, abstract, heading word, table of contents, key concepts, original title, tests & measures, mesh] |
| 16 | cryo-therapy.mp. [mp=title, abstract, heading word, table of contents, key concepts, original title, tests & measures, mesh] |
| 17 | cryotherapy.mp. [mp=title, abstract, heading word, table of contents, key concepts, original title, tests & measures, mesh] |
| 18 | (Percutaneous adj3 cryoablation).mp. [mp=title, abstract, heading word, table of contents, key concepts, original title, tests & measures, mesh] |
| 19 | Laparoscopic cryoablation.mp. [mp=title, abstract, heading word, table of contents, key concepts, original title, tests & measures, mesh] |
| 20 | Microwave ablation.mp. [mp=title, abstract, heading word, table of contents, key concepts, original title, tests & measures, mesh] |
| 21 | Radiofrequency ablation.mp. [mp=title, abstract, heading word, table of contents, key concepts, original title, tests & measures, mesh] |
| 22 | RFA.mp. [mp=title, abstract, heading word, table of contents, key concepts, original title, tests & measures, mesh] |
| 23 | mwa.mp. [mp=title, abstract, heading word, table of contents, key concepts, original title, tests & measures, mesh] |
| 24 | 1 or 2 or 3 or 4 or 5 or 6 or 7 or 8 or 9 or 10 or 11 or 12 or 13 or 14 or 15 or 16 or 17 or 18 or 19 or 20 or 21 or 22 or 23 |
| 25 | Quality of life.mp. or exp "Quality of Life"/ |
| 26 | qol.mp. |
| 27 | (Surveys and Questionnaires).mp. [mp=title, abstract, heading word, table of contents, key concepts, original title, tests & measures, mesh] |
| 28 | exp "Quality of Life Measures"/ or exp "Health Related Quality of Life"/ or Health related quality of life.mp. |
| 29 | Health-related quality of life.mp. |
| 30 | HRQoL.mp. |
| 31 | HR-QoL.mp. |
| 32 | exp Questionnaires/ or Quality of life questionnaire*.mp. |
| 33 | exp Surveys/ or SF-36.mp. |
| 34 | exp Psychometrics/ or Short form 36.mp. |
| 35 | SF-12.mp. |
| 36 | Short form 12.mp. |
| 37 | (European Organisation for Research and Treatment of Cancer).mp. [mp=title, abstract, heading word, table of contents, key concepts, original title, tests & measures, mesh] |
| 38 | EORTC.mp. |
| 39 | EORTC QlQ c-30.mp. |
| 40 | EQ-5D.mp. |
| 41 | EQ5D.mp. |
| 42 | Health Status.mp. or exp Health Status/ |
| 43 | EuroQoL.mp. |
| 44 | exp Patient Reported Outcome Measures/ or Patient reported outcome measures.mp. |
| 45 | pro.mp. |
| 46 | exp Well Being/ or Quality of wellbeing.mp. |
| 47 | exp Mental Health/ or Quality of well-being.mp. |
| 48 | Cancer Rehabilitation Evaluation System-Short form.mp. |
| 49 | CARES-SF.mp. |
| 50 | (Convalescence and recovery evaluation).mp. [mp=title, abstract, heading word, table of contents, key concepts, original title, tests & measures, mesh] |
| 51 | Functional assessment of cancer therapy-general.mp. |
| 52 | Functional assessment of cancer therapy-Kidney Symptom Index.mp. |
| 53 | Fact-g.mp. |
| 54 | FKSI.mp. |
| 55 | renal cell carcinoma symptom index.mp. |
| 56 | RCC-SI.mp. |
| 57 | 25 or 26 or 27 or 28 or 29 or 30 or 31 or 32 or 33 or 34 or 35 or 36 or 37 or 38 or 39 or 40 or 41 or 42 or 43 or 44 or 45 or 46 or 47 or 48 or 49 or 50 or 51 or 52 or 53 or 54 or 55 or 56 |
| 58 | ((Kidney or renal) adj3 (cancer* or carcinoma* or neoplasm* or tumo?r*)).mp. [mp=title, abstract, heading word, table of contents, key concepts, original title, tests & measures, mesh] |
| 59 | neoplasm.mp. or exp Neoplasms/ |
| 60 | kidney.mp. or exp Kidneys/ |
| 61 | 59 or 60 |
| 62 | locali?ed renal cell carcinoma.mp. [mp=title, abstract, heading word, table of contents, key concepts, original title, tests & measures, mesh] |
| 63 | 58 or 62 or 65 |
| 64 | 24 and 57 and 63 |

**Cochrane Library**

September 2020 n= 51

September 2021 n= 57

| #1 | MeSH descriptor: [Kidney Neoplasms] explode all trees |
| --- | --- |
| #2 | MeSH descriptor: [Carcinoma, Renal Cell] explode all trees |
| #3 | (kidney* near/3 tumor*):ti,ab,kw (Word variations have been searched) |
| #4 | ((kidney* near/3 neoplasm*)):ti,ab,kw (Word variations have been searched) |
| #5 | ((renal near/3 neoplasm*)):ti,ab,kw (Word variations have been searched) |
| #6 | ((renal near/3 tumor*)):ti,ab,kw (Word variations have been searched) |
| #7 | ((renal near/3 cancer*)):ti,ab,kw (Word variations have been searched) |
| #8 | ((kidney* near/3 cancer*)):ti,ab,kw (Word variations have been searched) |
| #9 | ((renal near/3 carcinoma*)):ti,ab,kw (Word variations have been searched) |
| #10 | ((kidney* near/3 carcinoma*)):ti,ab,kw (Word variations have been searched) |
| #11 | ((renal near/3 malign*)):ti,ab,kw (Word variations have been searched) |
| #12 | ((kidney* near/3 malign*)):ti,ab,kw (Word variations have been searched) |
| #13 | #1 OR #2 OR #3 OR #4 OR #5 OR #6 OR #7 OR #8 OR #9 OR #10 OR #11 OR #12 |
| #14 | MeSH descriptor: [Minimally Invasive Surgical Procedures] explode all trees |
| #15 | MeSH descriptor: [Cryotherapy] explode all trees |
| #16 | MeSH descriptor: [Robotic Surgical Procedures] 3 tree(s) exploded |
| #17 | MeSH descriptor: [Radiofrequency Therapy] explode all trees |
| #18 | MeSH descriptor: [Ablation Techniques] explode all trees |
| #19 | MeSH descriptor: [Laparoscopy] explode all trees |
| #20 | MeSH descriptor: [Hand-Assisted Laparoscopy] explode all trees |
| #21 | MeSH descriptor: [Robotic Surgical Procedures] 3 tree(s) exploded |
| #22 | (cryoablation* or cryo-ablation*):ti,ab,kw (Word variations have been searched) |
| #23 | (cryosurger* or cryo-surger*):ti,ab,kw (Word variations have been searched) |
| #24 | (cryotherap* or cryo-therap*):ti,ab,kw (Word variations have been searched) |
| #25 | (nephron sparing near/3 treatment*):ti,ab,kw (Word variations have been searched) |
| #26 | (nephron sparing near/3 surger*):ti,ab,kw (Word variations have been searched) |
| #27 | (nephron sparing near/3 procedure*):ti,ab,kw (Word variations have been searched) |
| #28 | (minimal* invasive near/3 surger*):ti,ab,kw (Word variations have been searched) |
| #29 | (minimally-invasive near/3 surger*):ti,ab,kw (Word variations have been searched) |
| #30 | (minimal* invasive near/3 procedure*):ti,ab,kw (Word variations have been searched) |
| #31 | (minimally-invasive near/3 procedure*):ti,ab,kw (Word variations have been searched) |
| #32 | ((minimal* invasive near/3 treatment*)):ti,ab,kw (Word variations have been searched) |
| #33 | ((minimally-invasive near/3 treatment*)):ti,ab,kw (Word variations have been searched) |
| #34 | (partial nephrectomy):ti,ab,kw (Word variations have been searched) |
| #35 | (robot* assisted near/3 surger*):ti,ab,kw (Word variations have been searched) |
| #36 | (robot-assisted near/3 surger*):ti,ab,kw (Word variations have been searched) |
| #37 | (robot-assisted near/3 procedure*):ti,ab,kw (Word variations have been searched) |
| #38 | (robot* assisted near/3 procedure*):ti,ab,kw (Word variations have been searched) |
| #39 | (robot* assisted near/3 treatment*):ti,ab,kw (Word variations have been searched) |
| #40 | (robot-assisted near/3 treatment*):ti,ab,kw (Word variations have been searched) |
| #41 | (laparoscop* near/3 partial nephrectom*):ti,ab,kw (Word variations have been searched) |
| #42 | (robot-assisted near/3 partial nephrectom*):ti,ab,kw (Word variations have been searched) |
| #43 | (robot* assisted near/3 partial nephrectom*):ti,ab,kw (Word variations have been searched) |
| #44 | (hand-assisted near/3 partial nephrectom*):ti,ab,kw (Word variations have been searched) |
| #45 | (laparoscop* near/3 surger*):ti,ab,kw (Word variations have been searched) |
| #46 | (laparoscop* near/3 treatment*):ti,ab,kw (Word variations have been searched) |
| #47 | (laparoscop* near/3 procedure*):ti,ab,kw (Word variations have been searched) |
| #48 | (Microwave ablation):ti,ab,kw (Word variations have been searched) |
| #49 | (Radiofrequency ablation):ti,ab,kw (Word variations have been searched) |
| #50 | (RFA):ti,ab,kw (Word variations have been searched) |
| #51 | (MWA):ti,ab,kw (Word variations have been searched) |
| #52 | #14 OR #15 OR #16 OR #17 OR #18 OR #19 OR #20 OR #21 OR #22 OR #23 OR #24 OR #25 OR #26 OR #27 OR #28 OR #29 OR #30 OR #31 OR #32 OR #33 OR #34 OR #35 OR #36 OR #37 OR #38 OR #39 OR #40 OR #41 OR #42 OR #43 OR #44 OR #45 OR #46 OR #47 OR #48 OR #49 OR #50 OR #51 |
| #53 | MeSH descriptor: [Quality of Life] explode all trees |
| #54 | MeSH descriptor: [Quality of Life] explode all trees |
| #55 | MeSH descriptor: [Surveys and Questionnaires] explode all trees |
| #56 | MeSH descriptor: [Patient Reported Outcome Measures] explode all trees |
| #57 | (quality of life OR QoL):ti,ab,kw (Word variations have been searched) |
| #58 | (health related quality of life):ti,ab,kw (Word variations have been searched) |
| #59 | Health-related quality of life |
| #60 | (HRQOL):ti,ab,kw (Word variations have been searched) |
| #61 | HR-QoL |
| #62 | (Quality of life questionnaire*):ti,ab,kw (Word variations have been searched) |
| #63 | (SF-36 OR Short form 36):ti,ab,kw (Word variations have been searched) |
| #64 | ("SF-12"):ti,ab,kw (Word variations have been searched) |
| #65 | ("SF-20"):ti,ab,kw (Word variations have been searched) |
| #66 | ("SF-36"):ti,ab,kw (Word variations have been searched) |
| #67 | ("Short Form 12"):ti,ab,kw (Word variations have been searched) |
| #68 | ("Short Form 8"):ti,ab,kw (Word variations have been searched) |
| #69 | ("SF-36 v2"):ti,ab,kw (Word variations have been searched) |
| #70 | ("SF-36v2"):ti,ab,kw (Word variations have been searched) |
| #71 | ("European Organisation for Research and Treatment of Cancer"):ti,ab,kw (Word variations have been searched) |
| #72 | ("Eortc"):ti,ab,kw (Word variations have been searched) |
| #73 | ("Eortc qlq c-30"):ti,ab,kw (Word variations have been searched) |
| #74 | ("EQ-5D"):ti,ab,kw (Word variations have been searched) |
| #75 | ("EQ5D"):ti,ab,kw (Word variations have been searched) |
| #76 | (euroqol):ti,ab,kw (Word variations have been searched) |
| #77 | MeSH descriptor: [Health Status] explode all trees |
| #78 | ("PRO"):ti,ab,kw (Word variations have been searched) |
| #79 | ("health status questionaire"):ti,ab,kw (Word variations have been searched) |
| #80 | ("health status"):ti,ab,kw (Word variations have been searched) |
| #81 | (Quality of well-being):ti,ab,kw (Word variations have been searched) |
| #82 | (Quality of wellbeing):ti,ab,kw (Word variations have been searched) |
| #83 | ("Cares-sf"):ti,ab,kw (Word variations have been searched) |
| #84 | (Fact-g):ti,ab,kw (Word variations have been searched) |
| #85 | ("Functional assessment of cancer therapy-Kidney Symptom Index"):ti,ab,kw (Word variations have been searched) |
| #86 | #53 OR #54 #55 OR #56 OR #57 OR #58 OR #59 OR #60 OR #61 OR #62 OR #63 OR #64 OR #65 OR #66 OR #67 OR #68 OR #69 OR #70 OR #71 OR #72 OR #73 OR #74 OR #75 OR #76 OR #77 OR #78 OR #79 OR #80 OR #81 OR #82 OR #83 OR #84 OR #85 |
| #87 | #13 AND #52 AND #86 |
